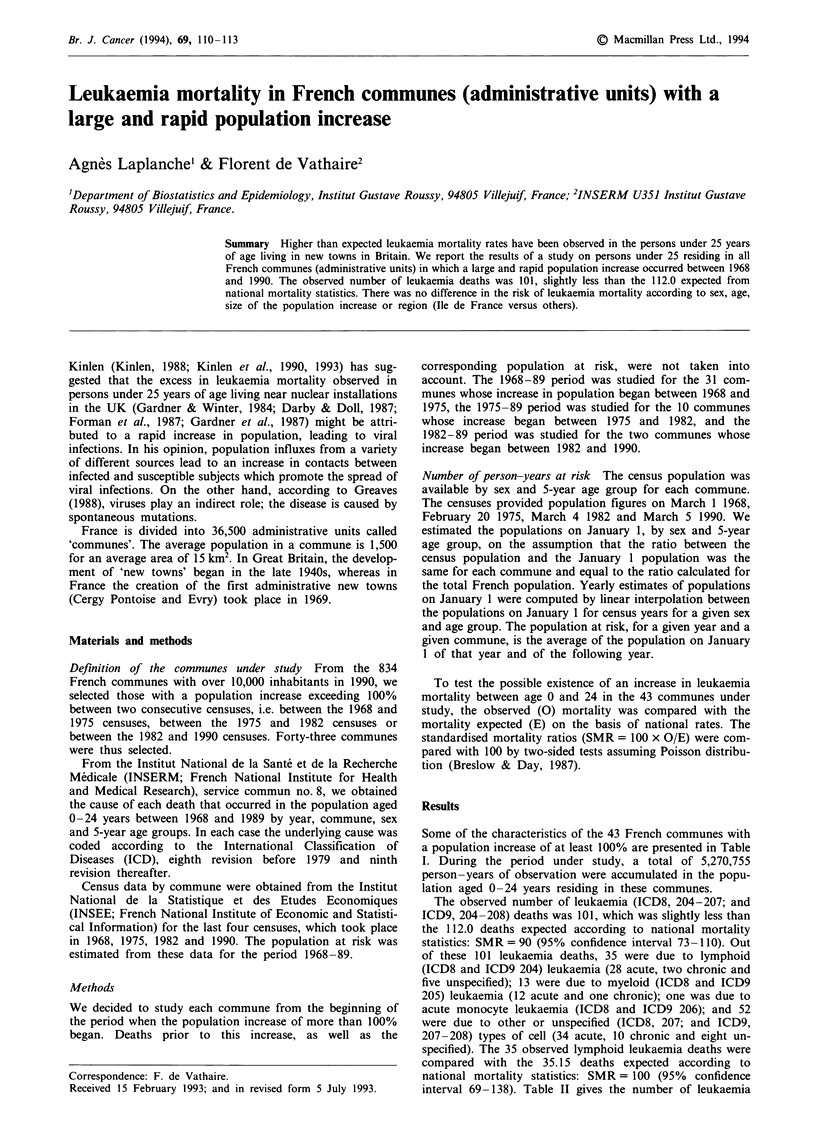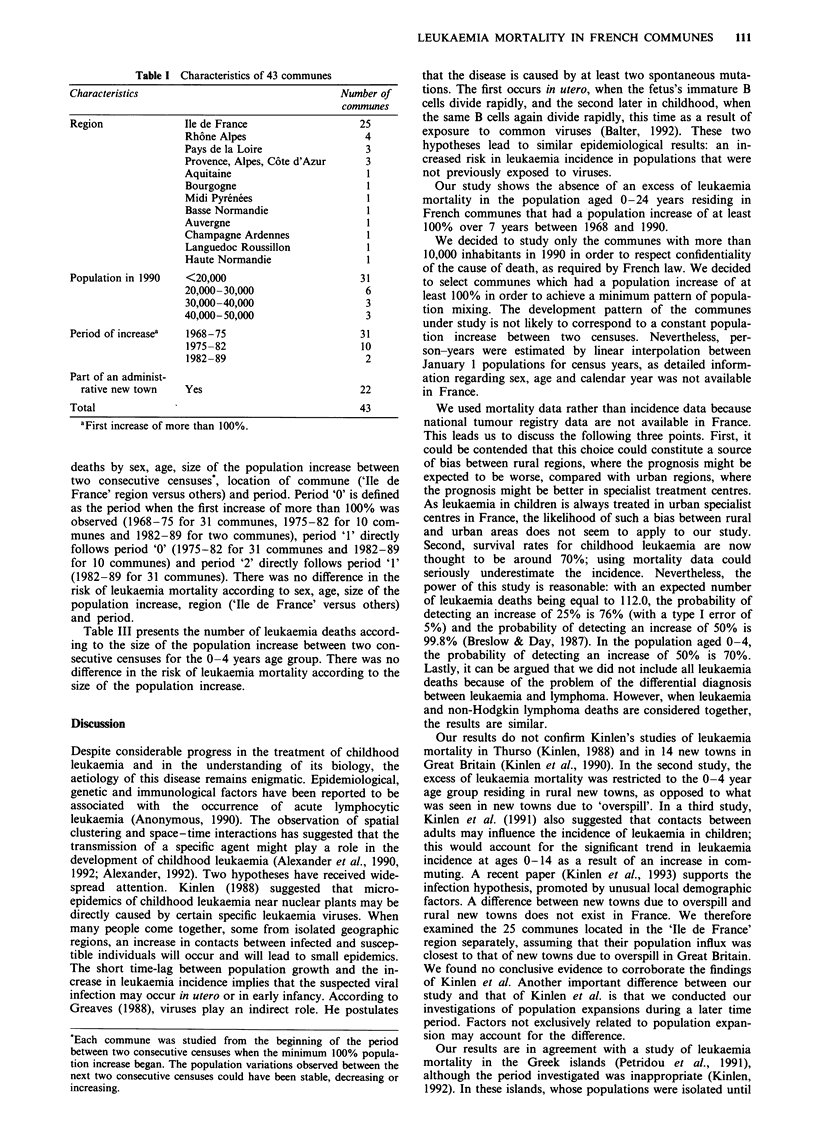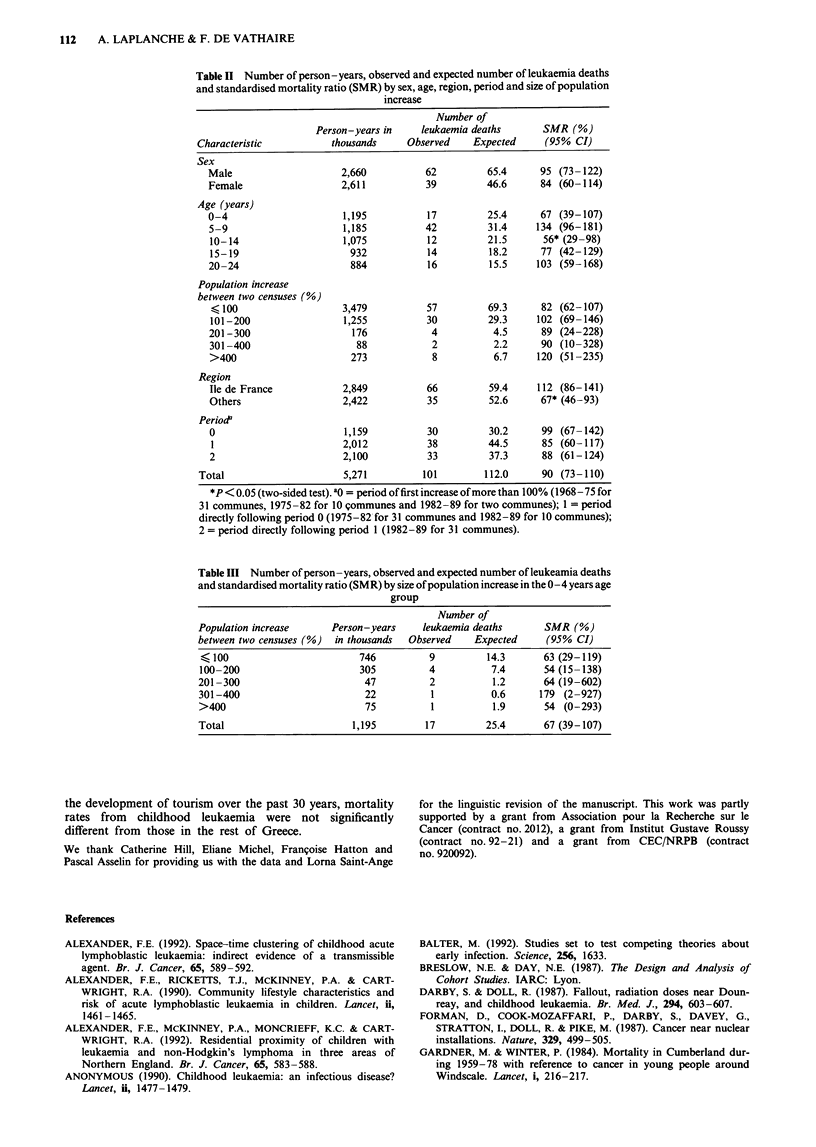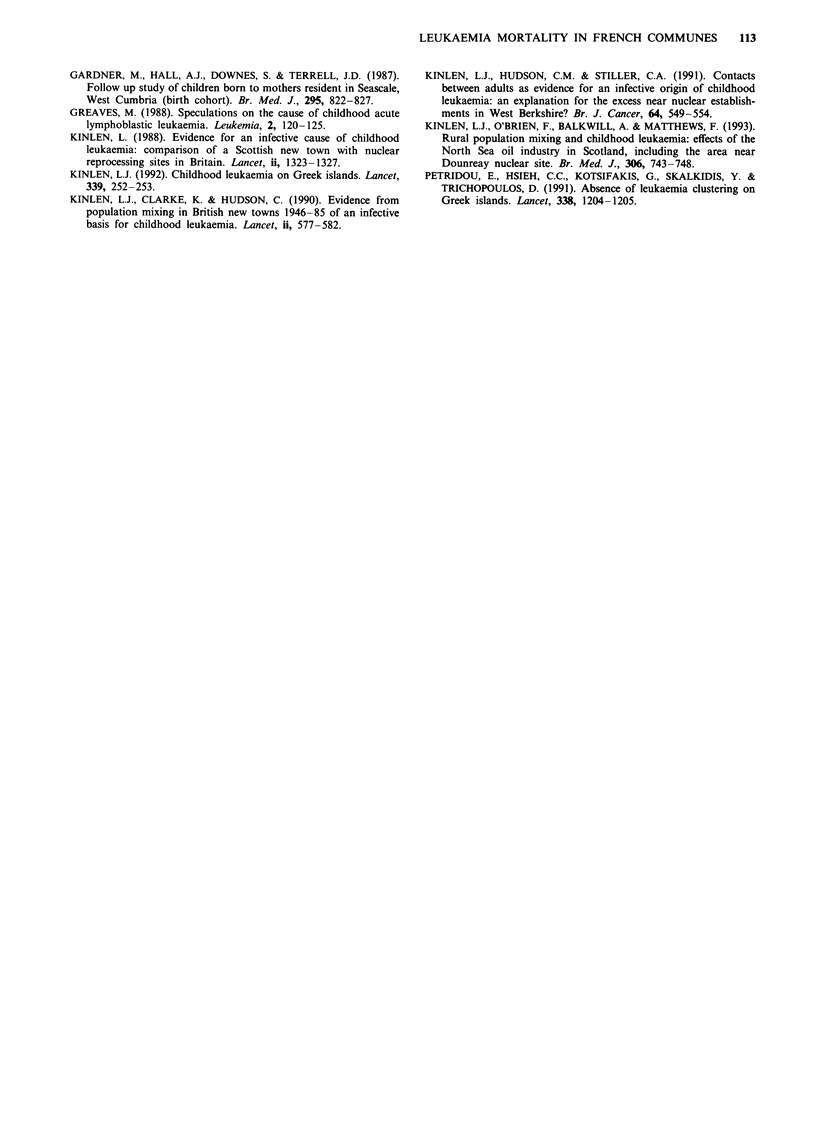# Leukaemia mortality in French communes (administrative units) with a large and rapid population increase.

**DOI:** 10.1038/bjc.1994.17

**Published:** 1994-01

**Authors:** A. Laplanche, F. de Vathaire

**Affiliations:** Department of Biostatistics and Epidemiology, Institut Gustave Roussy, Villejuif, France.

## Abstract

Higher than expected leukaemia mortality rates have been observed in the persons under 25 years of age living in new towns in Britain. We report the results of a study on persons under 25 residing in all French communes (administrative units) in which a large and rapid population increase occurred between 1968 and 1990. The observed number of leukaemia deaths was 101, slightly less than the 112.0 expected from national mortality statistics. There was no difference in the risk of leukaemia mortality according to sex, age, size of the population increase or region (Ile de France versus others).


Br. J. Cancer (1994), 69, 110-113 © Macmillan Press Ltd., 1994

### Leukaemia mortality in French communes (administrative units) with a large and rapid population increase

Agnès Laplanche[1] & Florent de Vathaire[2]

[1]*Department of Biostatistics and Epidemiology, Institut Gustave Roussy, 94805 Villejuif, France;* [2]*INSERM U351 Institut Gustave Roussy, 94805 Villejuif, France.*

**Summary** Higher than expected leukaemia mortality rates have been observed in the persons under 25 years of age living in new towns in Britain. We report the results of a study on persons under 25 residing in all French communes (administrative units) in which a large and rapid population increase occurred between 1968 and 1990. The observed number of leukaemia deaths was 101, slightly less than the 112.0 expected from national mortality statistics. There was no difference in the risk of leukaemia mortality according to sex, age, size of the population increase or region (Ile de France versus others).

Kinlen (Kinlen, 1988; Kinlen *et al.*, 1990, 1993) has suggested that the excess in leukaemia mortality observed in persons under 25 years of age living near nuclear installations in the UK (Gardner & Winter, 1984; Darby & Doll, 1987; Forman *et al.*, 1987; Gardner *et al.*, 1987) might be attributed to a rapid increase in population, leading to viral infections. In his opinion, population influxes from a variety of different sources lead to an increase in contacts between infected and susceptible subjects which promote the spread of viral infections. On the other hand, according to Greaves (1988), viruses play an indirect role; the disease is caused by spontaneous mutations.

France is divided into 36,500 administrative units called 'communes'. The average population in a commune is 1,500 for an average area of 15 $km^2$. In Great Britain, the development of 'new towns' began in the late 1940s, whereas in France the creation of the first administrative new towns (Cergy Pontoise and Evry) took place in 1969.

### Materials and methods

*Definition of the communes under study* From the 834 French communes with over 10,000 inhabitants in 1990, we selected those with a population increase exceeding 100% between two consecutive censuses, i.e. between the 1968 and 1975 censuses, between the 1975 and 1982 censuses or between the 1982 and 1990 censuses. Forty-three communes were thus selected.

From the Institut National de la Santé et de la Recherche Médicale (INSERM; French National Institute for Health and Medical Research), service commun no. 8, we obtained the cause of each death that occurred in the population aged 0-24 years between 1968 and 1989 by year, commune, sex and 5-year age groups. In each case the underlying cause was coded according to the International Classification of Diseases (ICD), eighth revision before 1979 and ninth revision thereafter.

Census data by commune were obtained from the Institut National de la Statistique et des Etudes Economiques (INSEE; French National Institute of Economic and Statistical Information) for the last four censuses, which took place in 1968, 1975, 1982 and 1990. The population at risk was estimated from these data for the period 1968-89.

### *Methods*

We decided to study each commune from the beginning of the period when the population increase of more than 100% began. Deaths prior to this increase, as well as the corresponding population at risk, were not taken into account. The 1968-89 period was studied for the 31 communes whose increase in population began between 1968 and 1975, the 1975-89 period was studied for the 10 communes whose increase began between 1975 and 1982, and the 1982-89 period was studied for the two communes whose increase began between 1982 and 1990.

*Number of person-years at risk* The census population was available by sex and 5-year age group for each commune. The censuses provided population figures on March 1 1968, February 20 1975, March 4 1982 and March 5 1990. We estimated the populations on January 1, by sex and 5-year age group, on the assumption that the ratio between the census population and the January 1 population was the same for each commune and equal to the ratio calculated for the total French population. Yearly estimates of populations on January 1 were computed by linear interpolation between the populations on January 1 for census years for a given sex and age group. The population at risk, for a given year and a given commune, is the average of the population on January 1 of that year and of the following year.

To test the possible existence of an increase in leukaemia mortality between age 0 and 24 in the 43 communes under study, the observed (O) mortality was compared with the mortality expected (E) on the basis of national rates. The standardised mortality ratios (SMR = 100 x O/E) were compared with 100 by two-sided tests assuming Poisson distribution (Breslow & Day, 1987).

### Results

Some of the characteristics of the 43 French communes with a population increase of at least 100% are presented in Table I. During the period under study, a total of 5,270,755 person-years of observation were accumulated in the population aged 0-24 years residing in these communes.

The observed number of leukaemia (ICD8, 204-207; and ICD9, 204-208) deaths was 101, which was slightly less than the 112.0 deaths expected according to national mortality statistics: SMR = 90 (95% confidence interval 73-110). Out of these 101 leukaemia deaths, 35 were due to lymphoid (ICD8 and ICD9 204) leukaemia (28 acute, two chronic and five unspecified); 13 were due to myeloid (ICD8 and ICD9 205) leukaemia (12 acute and one chronic); one was due to acute monocyte leukaemia (ICD8 and ICD9 206); and 52 were due to other or unspecified (ICD8, 207; and ICD9, 207-208) types of cell (34 acute, 10 chronic and eight unspecified). The 35 observed lymphoid leukaemia deaths were compared with the 35.15 deaths expected according to national mortality statistics: SMR = 100 (95% confidence interval 69-138). Table II gives the number of leukaemia

Correspondence: F. de Vathaire.
Received 15 February 1993; and in revised form 5 July 1993.

**Table I** Characteristics of 43 communes

| *Characteristics* | | *Number of communes* |
|---|---|---|
| Region | Ile de France | 25 |
| | Rhône Alpes | 4 |
| | Pays de la Loire | 3 |
| | Provence, Alpes, Côte d'Azur | 3 |
| | Aquitaine | 1 |
| | Bourgogne | 1 |
| | Midi Pyrénées | 1 |
| | Basse Normandie | 1 |
| | Auvergne | 1 |
| | Champagne Ardennes | 1 |
| | Languedoc Roussillon | 1 |
| | Haute Normandie | 1 |
| Population in 1990 | <20,000 | 31 |
| | 20,000–30,000 | 6 |
| | 30,000–40,000 | 3 |
| | 40,000–50,000 | 3 |
| Period of increase[a] | 1968–75 | 31 |
| | 1975–82 | 10 |
| | 1982–89 | 2 |
| Part of an administrative new town | Yes | 22 |
| Total | | 43 |

[a]First increase of more than 100%.

deaths by sex, age, size of the population increase between two consecutive censuses*, location of commune ('Ile de France' region versus others) and period. Period '0' is defined as the period when the first increase of more than 100% was observed (1968–75 for 31 communes, 1975–82 for 10 communes and 1982–89 for two communes), period '1' directly follows period '0' (1975–82 for 31 communes and 1982–89 for 10 communes) and period '2' directly follows period '1' (1982–89 for 31 communes). There was no difference in the risk of leukaemia mortality according to sex, age, size of the population increase, region ('Ile de France' versus others) and period.

Table III presents the number of leukaemia deaths according to the size of the population increase between two consecutive censuses for the 0–4 years age group. There was no difference in the risk of leukaemia mortality according to the size of the population increase.

### Discussion

Despite considerable progress in the treatment of childhood leukaemia and in the understanding of its biology, the aetiology of this disease remains enigmatic. Epidemiological, genetic and immunological factors have been reported to be associated with the occurrence of acute lymphocytic leukaemia (Anonymous, 1990). The observation of spatial clustering and space–time interactions has suggested that the transmission of a specific agent might play a role in the development of childhood leukaemia (Alexander *et al.*, 1990, 1992; Alexander, 1992). Two hypotheses have received widespread attention. Kinlen (1988) suggested that micro-epidemics of childhood leukaemia near nuclear plants may be directly caused by certain specific leukaemia viruses. When many people come together, some from isolated geographic regions, an increase in contacts between infected and susceptible individuals will occur and will lead to small epidemics. The short time-lag between population growth and the increase in leukaemia incidence implies that the suspected viral infection may occur *in utero* or in early infancy. According to Greaves (1988), viruses play an indirect role. He postulates that the disease is caused by at least two spontaneous mutations. The first occurs *in utero*, when the fetus's immature B cells divide rapidly, and the second later in childhood, when the same B cells again divide rapidly, this time as a result of exposure to common viruses (Balter, 1992). These two hypotheses lead to similar epidemiological results: an increased risk in leukaemia incidence in populations that were not previously exposed to viruses.

Our study shows the absence of an excess of leukaemia mortality in the population aged 0–24 years residing in French communes that had a population increase of at least 100% over 7 years between 1968 and 1990.

We decided to study only the communes with more than 10,000 inhabitants in 1990 in order to respect confidentiality of the cause of death, as required by French law. We decided to select communes which had a population increase of at least 100% in order to achieve a minimum pattern of population mixing. The development pattern of the communes under study is not likely to correspond to a constant population increase between two censuses. Nevertheless, person–years were estimated by linear interpolation between January 1 populations for census years, as detailed information regarding sex, age and calendar year was not available in France.

We used mortality data rather than incidence data because national tumour registry data are not available in France. This leads us to discuss the following three points. First, it could be contended that this choice could constitute a source of bias between rural regions, where the prognosis might be expected to be worse, compared with urban regions, where the prognosis might be better in specialist treatment centres. As leukaemia in children is always treated in urban specialist centres in France, the likelihood of such a bias between rural and urban areas does not seem to apply to our study. Second, survival rates for childhood leukaemia are now thought to be around 70%; using mortality data could seriously underestimate the incidence. Nevertheless, the power of this study is reasonable: with an expected number of leukaemia deaths being equal to 112.0, the probability of detecting an increase of 25% is 76% (with a type I error of 5%) and the probability of detecting an increase of 50% is 99.8% (Breslow & Day, 1987). In the population aged 0–4, the probability of detecting an increase of 50% is 70%. Lastly, it can be argued that we did not include all leukaemia deaths because of the problem of the differential diagnosis between leukaemia and lymphoma. However, when leukaemia and non-Hodgkin lymphoma deaths are considered together, the results are similar.

Our results do not confirm Kinlen's studies of leukaemia mortality in Thurso (Kinlen, 1988) and in 14 new towns in Great Britain (Kinlen *et al.*, 1990). In the second study, the excess of leukaemia mortality was restricted to the 0–4 year age group residing in rural new towns, as opposed to what was seen in new towns due to 'overspill'. In a third study, Kinlen *et al.* (1991) also suggested that contacts between adults may influence the incidence of leukaemia in children; this would account for the significant trend in leukaemia incidence at ages 0–14 as a result of an increase in commuting. A recent paper (Kinlen *et al.*, 1993) supports the infection hypothesis, promoted by unusual local demographic factors. A difference between new towns due to overspill and rural new towns does not exist in France. We therefore examined the 25 communes located in the 'Ile de France' region separately, assuming that their population influx was closest to that of new towns due to overspill in Great Britain. We found no conclusive evidence to corroborate the findings of Kinlen *et al.* Another important difference between our study and that of Kinlen *et al.* is that we conducted our investigations of population expansions during a later time period. Factors not exclusively related to population expansion may account for the difference.

Our results are in agreement with a study of leukaemia mortality in the Greek islands (Petridou *et al.*, 1991), although the period investigated was inappropriate (Kinlen, 1992). In these islands, whose populations were isolated until

*Each commune was studied from the beginning of the period between two consecutive censuses when the minimum 100% population increase began. The population variations observed between the next two consecutive censuses could have been stable, decreasing or increasing.

**Table II** Number of person–years, observed and expected number of leukaemia deaths and standardised mortality ratio (SMR) by sex, age, region, period and size of population increase

| *Characteristic* | *Person–years in thousands* | *Number of leukaemia deaths* *Observed* | *Expected* | *SMR (%) (95% CI)* |
|---|---|---|---|---|
| *Sex* | | | | |
| Male | 2,660 | 62 | 65.4 | 95 (73–122) |
| Female | 2,611 | 39 | 46.6 | 84 (60–114) |
| *Age (years)* | | | | |
| 0–4 | 1,195 | 17 | 25.4 | 67 (39–107) |
| 5–9 | 1,185 | 42 | 31.4 | 134 (96–181) |
| 10–14 | 1,075 | 12 | 21.5 | 56* (29–98) |
| 15–19 | 932 | 14 | 18.2 | 77 (42–129) |
| 20–24 | 884 | 16 | 15.5 | 103 (59–168) |
| *Population increase between two censuses (%)* | | | | |
| ⩽100 | 3,479 | 57 | 69.3 | 82 (62–107) |
| 101–200 | 1,255 | 30 | 29.3 | 102 (69–146) |
| 201–300 | 176 | 4 | 4.5 | 89 (24–228) |
| 301–400 | 88 | 2 | 2.2 | 90 (10–328) |
| >400 | 273 | 8 | 6.7 | 120 (51–235) |
| *Region* | | | | |
| Ile de France | 2,849 | 66 | 59.4 | 112 (86–141) |
| Others | 2,422 | 35 | 52.6 | 67* (46–93) |
| *Period*[a] | | | | |
| 0 | 1,159 | 30 | 30.2 | 99 (67–142) |
| 1 | 2,012 | 38 | 44.5 | 85 (60–117) |
| 2 | 2,100 | 33 | 37.3 | 88 (61–124) |
| Total | 5,271 | 101 | 112.0 | 90 (73–110) |

*$P < 0.05$ (two-sided test). [a]0 = period of first increase of more than 100% (1968–75 for 31 communes, 1975–82 for 10 çommunes and 1982–89 for two communes); 1 = period directly following period 0 (1975–82 for 31 communes and 1982–89 for 10 communes); 2 = period directly following period 1 (1982–89 for 31 communes).

**Table III** Number of person–years, observed and expected number of leukeamia deaths and standardised mortality ratio (SMR) by size of population increase in the 0–4 years age group

| *Population increase between two censuses (%)* | *Person–years in thousands* | *Number of leukaemia deaths* *Observed* | *Expected* | *SMR (%) (95% CI)* |
|---|---|---|---|---|
| ⩽100 | 746 | 9 | 14.3 | 63 (29–119) |
| 100–200 | 305 | 4 | 7.4 | 54 (15–138) |
| 201–300 | 47 | 2 | 1.2 | 64 (19–602) |
| 301–400 | 22 | 1 | 0.6 | 179 (2–927) |
| >400 | 75 | 1 | 1.9 | 54 (0–293) |
| Total | 1,195 | 17 | 25.4 | 67 (39–107) |

the development of tourism over the past 30 years, mortality rates from childhood leukaemia were not significantly different from those in the rest of Greece.

We thank Catherine Hill, Eliane Michel, Françoise Hatton and Pascal Asselin for providing us with the data and Lorna Saint-Ange for the linguistic revision of the manuscript. This work was partly supported by a grant from Association pour la Recherche sur le Cancer (contract no. 2012), a grant from Institut Gustave Roussy (contract no. 92–21) and a grant from CEC/NRPB (contract no. 920092).

### References

ALEXANDER, F.E. (1992). Space–time clustering of childhood acute lymphoblastic leukaemia: indirect evidence of a transmissible agent. *Br. J. Cancer*, **65**, 589–592.

ALEXANDER, F.E., RICKETTS, T.J., McKINNEY, P.A. & CARTWRIGHT, R.A. (1990). Community lifestyle characteristics and risk of acute lymphoblastic leukaemia in children. *Lancet*, **ii**, 1461–1465.

ALEXANDER, F.E., McKINNEY, P.A., MONCRIEFF, K.C. & CARTWRIGHT, R.A. (1992). Residential proximity of children with leukaemia and non-Hodgkin's lymphoma in three areas of Northern England. *Br. J. Cancer*, **65**, 583–588.

ANONYMOUS (1990). Childhood leukaemia: an infectious disease? *Lancet*, **ii**, 1477–1479.

BALTER, M. (1992). Studies set to test competing theories about early infection. *Science*, **256**, 1633.

BRESLOW, N.E. & DAY, N.E. (1987). *The Design and Analysis of Cohort Studies*. IARC: Lyon.

DARBY, S. & DOLL, R. (1987). Fallout, radiation doses near Dounreay, and childhood leukaemia. *Br. Med. J.*, **294**, 603–607.

FORMAN, D., COOK-MOZAFFARI, P., DARBY, S., DAVEY, G., STRATTON, I., DOLL, R. & PIKE, M. (1987). Cancer near nuclear installations. *Nature*, **329**, 499–505.

GARDNER, M. & WINTER, P. (1984). Mortality in Cumberland during 1959–78 with reference to cancer in young people around Windscale. *Lancet*, **i**, 216–217.

GARDNER, M., HALL, A.J., DOWNES, S. & TERRELL, J.D. (1987). Follow up study of children born to mothers resident in Seascale, West Cumbria (birth cohort). *Br. Med. J.*, **295**, 822–827.

GREAVES, M. (1988). Speculations on the cause of childhood acute lymphoblastic leukaemia. *Leukemia*, **2**, 120–125.

KINLEN, L. (1988). Evidence for an infective cause of childhood leukaemia: comparison of a Scottish new town with nuclear reprocessing sites in Britain. *Lancet*, **ii**, 1323–1327.

KINLEN, L.J. (1992). Childhood leukaemia on Greek islands. *Lancet*, **339**, 252–253.

KINLEN, L.J., CLARKE, K. & HUDSON, C. (1990). Evidence from population mixing in British new towns 1946–85 of an infective basis for childhood leukaemia. *Lancet*, **ii**, 577–582.

KINLEN, L.J., HUDSON, C.M. & STILLER, C.A. (1991). Contacts between adults as evidence for an infective origin of childhood leukaemia: an explanation for the excess near nuclear establishments in West Berkshire? *Br. J. Cancer*, **64**, 549–554.

KINLEN, L.J., O'BRIEN, F., BALKWILL, A. & MATTHEWS, F. (1993). Rural population mixing and childhood leukaemia: effects of the North Sea oil industry in Scotland, including the area near Dounreay nuclear site. *Br. Med. J.*, **306**, 743–748.

PETRIDOU, E., HSIEH, C.C., KOTSIFAKIS, G., SKALKIDIS, Y. & TRICHOPOULOS, D. (1991). Absence of leukaemia clustering on Greek islands. *Lancet*, **338**, 1204–1205.